# Multi-omics analysis of the role of muskmelon mitochondria in Si-induced resistance mechanisms

**DOI:** 10.3389/fpls.2026.1820516

**Published:** 2026-06-12

**Authors:** Chenglong Zhao, Liang Lyu, Yuchao Ning, Yawen Luo, Xining He, Yuyan Yang, Yingjie Sun, Wen Ma

**Affiliations:** 1 College of Biological and Pharmaceutical Engineering, Lanzhou Jiaotong University, Lanzhou, China; 2 Department of Foreign Languages, University of Chinese Academy of Sciences, Beijing, China; 3 School of Biomedical Sciences, The Chinese University of Hong Kong, Hong Kong, Hong Kong SAR, China

**Keywords:** *Cucumis melo*, DNA methylomics, induced resistance, multi-omics, silicon, *Trichothecium roseum*

## Abstract

Post-harvest senescence and fungal infection, particularly Pink Mold Rot caused by *Trichothecium roseum*, significantly compromise the storage quality and economic value of muskmelon (*Cucumis melo* cv. Yujinxiang). While Silicon (Si) application is recognized as an effective strategy for enhancing post-harvest disease resistance, the molecular mechanisms driving this resistance—specifically the interplay between DNA methylation, transcriptional regulation, and mitochondrial function—remain largely uncharacterized. This study employed an integrated multi-omics approach, combining Whole-Genome Bisulfite Sequencing (WGBS), RNA sequencing (RNA-*seq*), and physiological assays, to elucidate the regulatory networks induced by Si treatment in T. roseum-inoculated muskmelons. Physiologically, Si treatment significantly alleviated oxidative stress and maintained mitochondrial energy status, evidenced by elevated ATP levels and increased Ca^2+^-ATPase activity compared to untreated controls. At the epigenomic level, we identified a distinct, context-specific enhancement of DNA methylation induced by Si. Notably, the CG context within the upstream 2 kilobases (up2k) regions exhibited the most significant response to treatment, surpassing changes in CHG and CHH contexts. Integrative analysis of the methylome and transcriptome revealed that this observed promoter hypermethylation correlates with the fine-tuning of gene expression related to energy metabolism and defense. Rather than simple repression, this epigenetic modification appears to stabilize transcriptional responses, potentially preventing the metabolic energy drain associated with hyper-immune responses or delaying the activation of senescence-associated genes. These findings establish a novel mechanistic link between epigenetic modification, and cellular energy metabolism. Ultimately, this research provides a theoretical basis for the utilization of Si as a post-harvest treatment to extend the shelf life of muskmelons by reinforcing mitochondrial function via epigenetic regulation.

## 1 Introduction

Muskmelon (*Cucumis melo* L) is a widely cultivated economic crop valued for its nutritional quality and unique flavor (Lastochkina et al., 2019). However, due to its climacteric nature and high water content, the fruit is highly susceptible to post-harvest senescence and fungal pathogens, which significantly restricts its shelf life and marketability (Bi et al., 2006). Among the various post-harvest diseases, Pink Mold Rot, caused by *Trichothecium roseum* (*T. roseum*), poses a severe threat to the melon industry, leading to rapid tissue decay and mycotoxin contamination (Lyu et al., 2025b). Therefore, developing effective, safe, and sustainable strategies to enhance the innate resistance of muskmelons against such pathogens is a critical priority for post-harvest biology.

Phenylpropanoid metabolism is a crucial metabolic pathway that is rapidly activated in plants during defense against pathogen infection, in which phenylalanine ammonia-lyase (PAL) and peroxidase (POD) play central roles. As the rate-limiting enzyme of phenylpropanoid metabolism, PAL catalyzes the conversion of phenylalanine to trans-cinnamic acid, thereby providing precursors for the subsequent biosynthesis of lignin, flavonoids, and phenolic compounds, which in turn reinforces cell wall architecture and enhances plant disease resistance (Dong and Lin, 2021). POD participates in lignin polymerization and cell wall cross-linking processes, while also influencing defense signal transduction and localized cell death responses through the modulation of reactive oxygen species (ROS) levels. Therefore, elucidating the synergistic regulatory mechanisms of PAL and POD in phenylpropanoid metabolism holds significant theoretical value and practical implications for advancing the understanding of plant immune responses and enhancing crop disease resistance.

Recent research has increasingly emphasized the metabolic regulation of fruit immunity, with particular attention to the role of early defense responses closely associated with mitochondrial metabolism. Mitochondria are now recognized as key signaling hubs governing both energy supply and ROS production (Lyu et al., 2019). In parallel, exogenous applications such as silicon (Si) have been shown to be effective, primarily through the reinforcement of the induction of systemic resistance (Hadizadeh et al., 2024). Si, widely recognized as a beneficial element for plant growth, has been shown to play a pivotal role in alleviating biotic and abiotic stresses. Previous studies have demonstrated that exogenous Si treatment can induce resistance against fungal pathogens by reinforcing cell walls and activating defense-related enzymatic systems (Prusky and Romanazzi, 2023). In the context of muskmelons, recent research indicates that early defense responses are closely linked to mitochondrial energy metabolism (Lyu et al., 2019). The mitochondrial respiratory metabolic pathway serves as a crucial hub for integrating energy provision and disease resistance responses in plants, and plays a significant regulatory role in the immune response induced by pathogen infection. Pathogen stimulation can induce respiratory chain reprogramming and an elevated energy demand, wherein succinate dehydrogenase (SDH) and cytochrome c oxidase (CCO) participate in the tricarboxylic acid (TCA) cycle and the terminal oxidation process of the electron transport chain, respectively, thereby maintaining ATP synthesis and metabolic homeostasis while providing energy and reducing power to support defense responses (Zhou et al., 2022). Mitochondria serve as the hub for energy production and redox regulation, and the maintenance of adequate ATP levels and ATPase activity—such as Ca^2+^-ATPase and H^+^-ATPase—is essential for sustaining cellular homeostasis and fueling defense reactions during pathogen attack (Lu et al., 2021). While the physiological impacts of Si on energy metabolism have been documented, the upstream molecular mechanisms regulating these processes remain largely unexplored.

Epigenetic modifications, particularly DNA methylation, have emerged as key regulators of gene expression in response to environmental stimuli and fruit development (Ullah et al., 2022). In horticultural crops such as tomato (Zhou et al., 2023), kiwifruit (Zhang et al., 2020), and strawberry (Cai et al., 2021), DNA methylation dynamics have been associated with ripening and disease resistance. However, the interplay between DNA methylation, transcriptional regulation, and mitochondrial function in Si-induced resistance in muskmelons has not been elucidated.

This study employs a multi-omics approach, integrating WGBS and RNA-*seq*, to investigate the molecular mechanisms underlying Si-induced resistance in *T. roseum*-inoculated muskmelons. Specifically, we focused on the alterations in DNA methylation patterns and their correlation with transcriptomic changes and mitochondrial energy metabolism parameters, including ATP content and ATPase activities. By deciphering the epigenetic regulation of mitochondrial function, this research aims to provide novel insights into the regulatory networks of Si-induced resistance mechanisms.

## 2 Materials and methods

### 2.1 Fruit material and *T. roseum* inoculation

Muskmelons (*Cucumis melo* cv. Yujinxiang) with no injuries and infectionswere harvested in Lanzhou City, located in Gansu Province, Northwest China. The fruits were harvested at 40 days after anthesis, selecting specimens of uniform size and shape, free from any insect damage and infection. The fruits were packed in paperboard boxes and transported to the laboratory for storage until the next stage of use. The room temperature was maintained at 22 ± 2 °C, with relative humidity kept between 55-60%. *T. roseum* link was obtained from infected fruits and preserved in -80 °C until the next stage of use. For each group, 12 fruits were used, as well as three independent biological replicates, each comprising four muskmelon fruits. The method of muskmelon fruit inoculated with fungus was according to the description of (Li et al., 2012).

### 2.2 Fruit treatments and sample collection

The approach described by (Li et al., 2012) was employed as the standardized procedure for administering Si treatment and performing artificial inoculation. The Si solution was formulated at a concentration of 100 mM by dissolving the supplied compound (27% SiO_2_, Sigma Aldrich) in distilled water, which served as the solvent. Following the Si treatment, artificial inoculation was subsequently carried out after a 24 hours post-inoculation (hpi) interval. Fruit decay was quantified at designated time points (1, 3, 5, and 7 day)following inoculation. Sample collection was performed as per the method outlined by (Li et al., 2012). Following the protocol established by (Li et al., 2012), the natural incidence was quantified every 48 h during storage under controlled conditions (22 ± 2°C), continuing until day 13. Fruits tissues with a depth of 1.5 cm were taken at the cross area of disease lesion and health tissue using a sterilized cork borer with 70% ethanol. Disease quantification was based on the established criterion of a mold spot diameter> 1 cm on the fruit surface. The experimental design included three independent replications, with each treatment comprising 12 muskmelons.

### 2.3 Determination of the activities of ROS, SDH, CCO, H^+^-ATPase, Ca^2+^-ATPase and ATP in fruit

The emergence of O_2_^•−^ followed the method described by (Elstner and Heupel, 1976) for measurement. Homogenates were prepared by grinding five grams of flesh tissue in 5 mL of 50 mmol L^-1^ phosphate buffer (pH 7.8), using a sterile mortar and pestle. The resulting homogenate was subsequently centrifuged at 10, 000 × g for 20 min at 4 °C. The supernatant was assayed for O_2_**^•−^** production by reference to a standard curve. The values are reported in units of nmol [NO_2_] g^−1^ FW min^−1^.The H_2_O_2_ content of the fungal-inoculated and control muskmelon fruits was assayed employing the procedure established by (Patterson et al., 1984). The H_2_O_2_ content in flesh tissue was determined using a spectrophotometric assay based on the absorbance of the titanium peroxide complex at 410 nm. H_2_O_2_ levels were quantified against a standard curve generated from serially diluted H_2_O_2_ standards, with results reported in µmol g^-1^ FW.

Extraction of crude mitochondria was performed according to (Jin et al., 2013), and their subsequent quality was assessed (Jin et al., 2013). Homogenization of frozen samples was conducted with 10 mL of extracting buffer maintained at 4 °C. The homogenate was clarified by filtration through gauze and subsequent centrifugation. The discarded pellet was followed by a second centrifugation of the supernatant. The final pellet was collected and resuspended in 2 mL of washing buffer to prepare for enzyme assays.

SDH activity was determined employing the protocol established by (Wang et al., 2022). To initiate the reaction, 0.2 M potassium phosphate buffer (pH 7.4, supplemented with 0.2 M sodium succinate) and 0.9mM dichlorophenol indophenol sodium were added to the mitochondrial extract that had been pre-incubated at 30 °C for 5 min. Following this, the reaction mixture was supplemented with 0.1 mL of phenazine methosulfate to commence the reaction. Enzyme activity was quantified in terms of units, where one unit of SDH activity is defined as the amount that causes an absorbance change of 0.01 per minute at A600 in the standard assay mixture.

The activity of CCO was subsequently determined by the method of (Li X. et al., 2023; Veitch et al., 1992) after the reaction mixture was incubated at 30 °C for 10 min. The reaction mixture, containing crude mitochondrial extracts and 0.04% cytochrome c, was incubated at 37 °C for 2 min. Thereafter, 0.4% dimethyl phenylene diamine was added, and the mixture was reincubated at 37 °C for 10 min. CCO activity was quantified as units (U) per mg protein, with one unit (U) representing a ΔA550 of 0.01 per second under the assay conditions.All assays were performed in triplicate.

The purity of the isolated mitochondria was verified by measuring the activity of marker enzymes. The high activities of succinate dehydrogenase (SDH) and cytochrome c oxidase (CCO), which are mitochondrial inner membrane markers, combined with the differential centrifugation steps (Jin et al., 2013), ensured the enrichment of mitochondrial fractions and minimized contamination from other organelles.

H^+^-ATPase activity was assayed in a reaction mixture consisting of the mitochondrial extract and an extraction buffer (pH 8.0, supplemented with 0.1 mM ammonium molybdate, 0.1 mM Na_3_VO_4_, 3 mM MgSO_4_, 50 mM NaNO_3_, and 50 mM KCl). The reaction was commenced with the introduction of 30 mM ATP-HCl solution (pH 8.0). After proceeding for 20 min at 37 °C, the reaction was quenched by the addition of an equal volume of 55% (w/v) trichloroacetic acid. The assay for Ca^2+^-ATPase employed an extraction buffer identical in composition to that used for H^+^-ATPase, except for the absence of MgSO_4_. For both enzymes, one unit of activity was defined as the quantity that liberated 1 mmol of phosphorus per hour (Ge et al., 2017), with the liberated phosphorus quantified by absorbance at 660 nm (Jin et al., 2014). The data presented are the mean values from three independent replicates per sample (Jin et al., 2014). To ensure the specificity of mitochondrial H^+^-ATPase and Ca²^+^-ATPase assays, selective inhibitors were added to the reaction mixture to exclude interference from other ATPases. Sodium orthovanadate (0.1 mM Na_3_VO_4_) was used to inhibit plasma membrane P-type ATPases, sodium nitrate (50 mM NaNO_3_) was included to inhibit vacuolar V-type ATPases, and ammonium molybdate (0.1 mM) was used to suppress non-specific acid phosphatase activity (Jin et al., 2014; Ge et al., 2017). Thus, the liberated phosphorus measured directly reflected the activity of the mitochondrial-targeted enzymes.

The 10% (w/v) homogenate was prepared by boiling the tissue in double-distilled water for exactly 10 minutes. This duration was chosen to ensure the immediate denaturation of ATP-degrading enzymes (such as ATPases and phosphatases) while minimizing the thermal hydrolysis of nucleotides. Following boiling, the extracts were rapidly cooled on ice and centrifuged at 10, 000 × g for 15 min at 4 °C to maintain the stability of the ATP pool (Lyu et al., 2019).” The ATP content was quantified using a bioluminescence assay kit based on the firefly luciferase-luciferin system (Nanjing Jiancheng Bioengineering Institute, China). The reaction involves the luciferase-catalyzed oxidation of luciferin in the presence of ATP and Mg^2+^, producing light proportional to the ATP concentration. To manage potential interference from endogenous pigments or inhibitors, the supernatant was appropriately diluted, and a specific Tris-Acetate buffer (40 mM, pH 8.0) was used to stabilize the bioluminescence signal.” A standard calibration curve was generated using a series of known ATP standards (ranging from 0.01 to 1 μmol L^−1^). The bioluminescence intensity was measured using a luminometer (or microplate reader). The final ATP content was calculated based on the standard curve and expressed as nmol g^−1^ fresh weight (FW)”.

### 2.4 Construction of methyl−*seq* libraries and data processing

Genomic DNA purification from muskmelon was performed using the E.Z.N.A.^®^ Plant DNA Kit (Omega Bio-tek, Norcross, GA, USA), following the manufacturer’s protocol. Only DNA specimens that passed stringent quality control—exhibiting an OD 260/280 ratio within the optimal range of 1.8 to 2.0 and a concentration yielding > 6 µg of DNA—were subjected to fragment library preparation. Library preparation for methyl-Seq employed the established protocol of (Bhat et al., 2020) and (Sun et al., 2019). Three biological replicates per sample were used. Shanghai OE Biotech Co., Ltd. in Shanghai, China performed WGBS. Sequencing libraries were constructed according to the directional WGBS protocol provided by OE Biotech. Briefly, genomic DNA was subjected to sonication, yielding fragments ranging from 100 to 300 bp. Bisulfite treatment was carried out using the EZ DNA methylation Gold Kit (ZYMO, Mexico City, Mexico). The WGBS analysis was performed employing 150 bp paired-end sequencing on an Illumina NovaSeq 6000 system, with a mean depth of coverage of 12 × on across the genome.

Preprocessing of raw sequencing data was employed by fastp (v0.20.0), comprising the following steps: low-complexity filtering, 3′-end polyX trimming, and base correction for overlapping regions (Chen et al., 2018) High-quality bisulfite-seq reads were aligned to the muskmelon (*Cucumis melo.* cv) reference assembly using BSMAP (Liu et al., 2015). Identification of differentially methylated regions (DMRs) was carried out with the R package MethylKit (Zhong et al., 2013). Sequence motif analysis and visualization of the resulting DMRs were then performed using ggseqlogo (Wagih, 2017). Enrichment analysis for Gene Ontology terms was conducted with the clusterProfiler package (v3.14.0) in R. GO enrichment analysis was conducted using the clusterProfiler package in R. Statistically significant GO terms were identified with a significance threshold set at a q-value less than 0.05 (Yu et al., 2012).

### 2.5 RNA sequencing and data analysis

To investigate the transcriptional changes in response to inoculation, RNA-*seq* was conducted on muskmelon fruits at the 24 hours post-inoculation (hpi). The selection of this specific time point was guided by the methylation dynamics observed in our prior WGBS data. The experimental design included three biological replicates for both the control and Si-treated groups. Each replicate contained a pool of 12 muskmelon fruits, and total RNA was separately extracted from each pooled sample. Total RNA was purified from samples using TRIzol reagent (Invitrogen, Carlsbad, CA, USA) following the supplier’s guidelines to ensure RNA quality. Subsequently, mRNA was isolated, fragmented, and reverse-transcribed for the generation of sequencing-ready cDNA libraries (Xu et al., 2016). High-throughput sequencing of the cDNA library was commercially obtained from Genedenovo Biotechnology Co., Ltd. (Guangzhou, China) using their Illumina platform. The interplay between DNA methylation dynamics and transcriptomic changes was systematically examined following the methodological framework of (Xu et al., 2016). High-throughput sequencing was performed on the Illumina NovaSeq 6000 platform with 150 bp paired-end (PE150) reads. Each sample yielded a mean depth of approximately 20 million raw reads.” Libraries were constructed cDNA libraries.

### 2.6 Proteome analysis and data analysis

Tissues for proteomic analysis were harvested in parallel from the corresponding replicates employed for RNA-*seq* (three biological replicates per condition) (Schiller et al., 2025). Tissues were cryogenically ground to a fine powder under liquid nitrogen and then resuspended in 400 µL of protein extraction buffer (4% SDS, 100 mM Tris-HCl, 100 mM DTT, pH 7.6). Subsequently, The total protein was extracted from the samples. One aliquot was used for protein concentration quantification and SDS-PAGE analysis, whereas the remaining portion was subjected to trypsin digestion and labeling. Equal amounts of each labeled sample were subsequently combined, followed by chromatographic separation and LC-MS/MS (Q Exactive HF-X) analysis for protein identification and quantification (Candiano et al., 2004). The optical density (OD) at 562 nm was quantified on a Multiskan FC microplate reader (Thermo Scientific, Waltham, MA, USA).

The core bioinformatics workflow for TMT (TMT 10) proteomics integrates several key analytical steps. Initially, proteins are filtered for reliable quantification and statistical significance to define a set of differential expression. This protein set is then subjected to a multi-faceted functional interrogation, including GO term enrichment, pathway mapping, and protein-protein interaction network construction (Wiśniewski et al., 2009).

### 2.7 Statistics analysis

All analysis was conducted using SPSS 17.0 (SPSS, Inc., Chicago, IL). The data were presented as mean value ± standard errors (SE) following the application of Duncan’s multiple range tests within a two-way analysis of variance (ANOVA) (treatment × time).

## 3 Results

### 3.1 Si treatment inhibited *T. roseum* infection in muskmelon fruits

**Figure 1:**
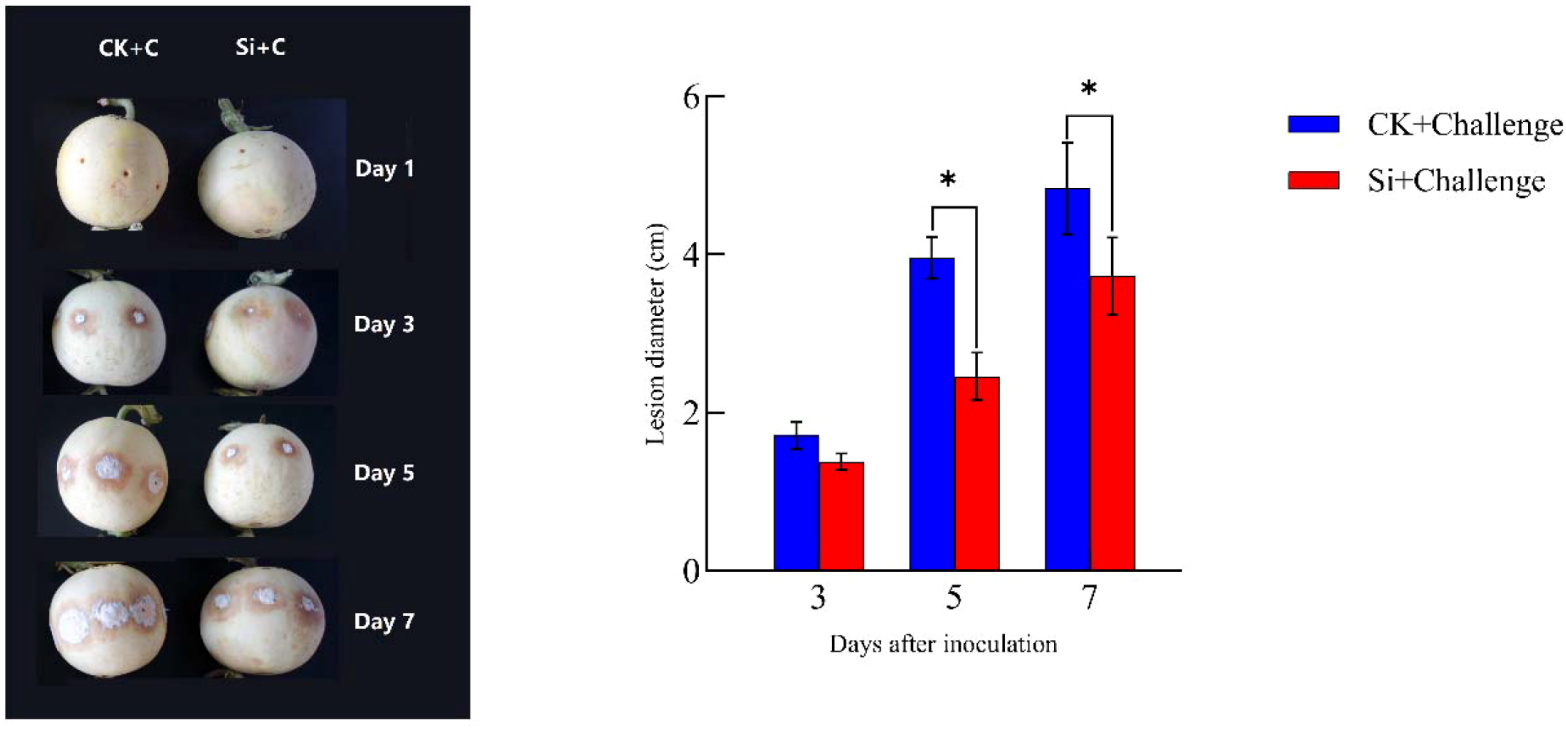
Phenotypic comparison of fruit lesion development in infected control and Si-treated groups; *, *p* < 0.05; CK + C, control check and pathogen challenge; Si + C, Si treatment and pathogen challenge. Three independent biological replicates were set (n = 3), and each replicate contained four muskmelon fruits (12 fruits in total for per treatment group).

Marked phenotypic shifts were concurrently observed in the Si-treated (Si+C) and infected control (CK+C) groups commencing 3 days preceding inoculation. The Si-treated group demonstrated a significant inhibitory effect on the progression of fruit lesion area when compared to the infected control (**Figure 1**). The pathogenicity assay confirmed that *T. roseum* inoculation elicited defined necrotic lesions and decay symptoms on muskmelon fruit surfaces. Contrary to the increased natural disease incidence observed in both groups throughout storage, Si application effectively attenuated this increase relative to the control.

### 3.2 Si treatment enhanced ROS production

**Figure 2:**
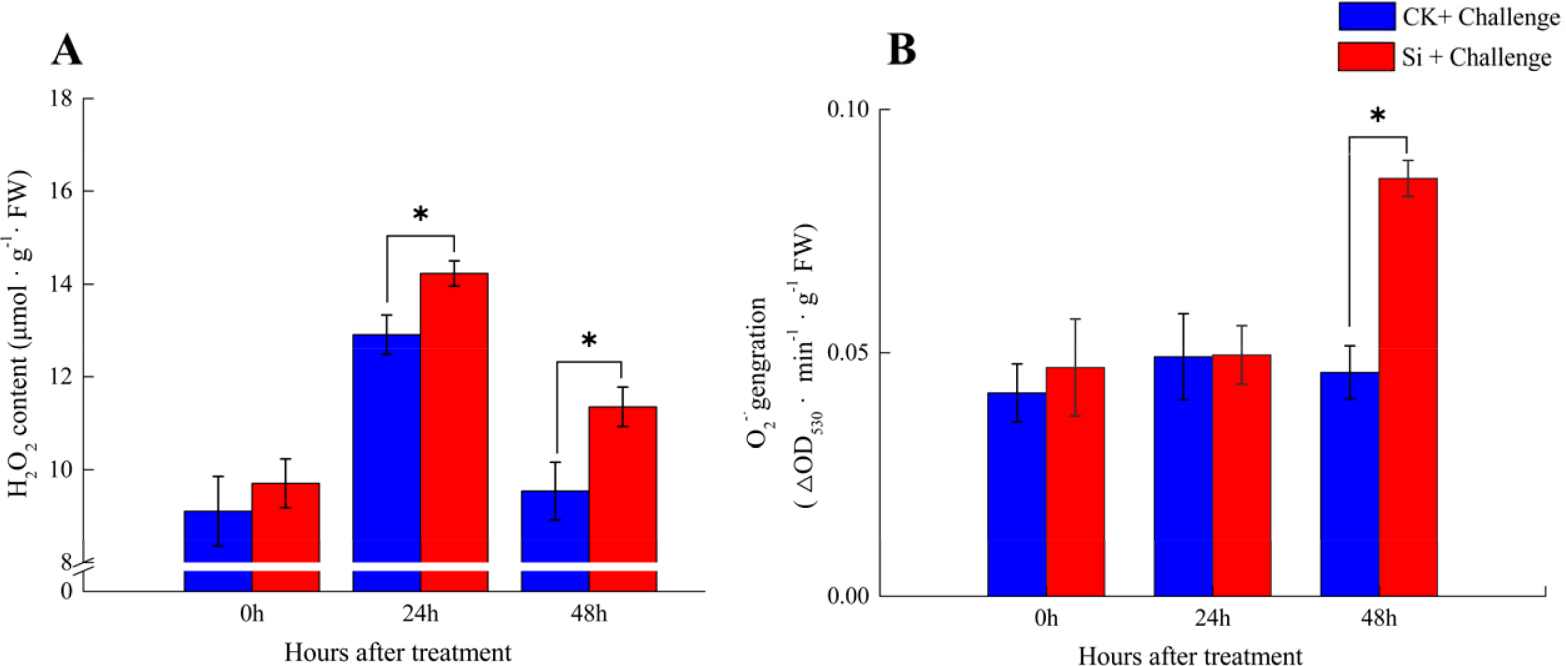
O_2_^•−^ generation rate **(A)**, H_2_O_2_ content **(B)** of muskmelon fruit; *, *p* < 0.05; CK + C, control check and pathogen challenge; Si + C, Si treatment and pathogen challenge. (Data represent mean ± SEM from n = 3 independent biological replicates).

A marked induction of H_2_O_2_ was observed in the the junction of decay and healthy regions following Si treatment, with a distinct peak occurring at 24 hpi which increased by 10.26% compared to the CK+C (*p* < 0.05). Si treatment resulted in an 18.95% higher H_2_O_2_ content at 48 hpi relative to the infected control group (**Figure 2A**). Additionally, although the magnitude of increase was limited to 0.0125%, the O_2_**^•−^** production demonstrated a statistically significant peak at 24 hpi compared to the infected group (**Figure 2B**, *p* < 0.05).

### 3.3 Effects of si treatment on the activity of respiratory electron transport chain enzymes and ATP production

**Figure 3:**
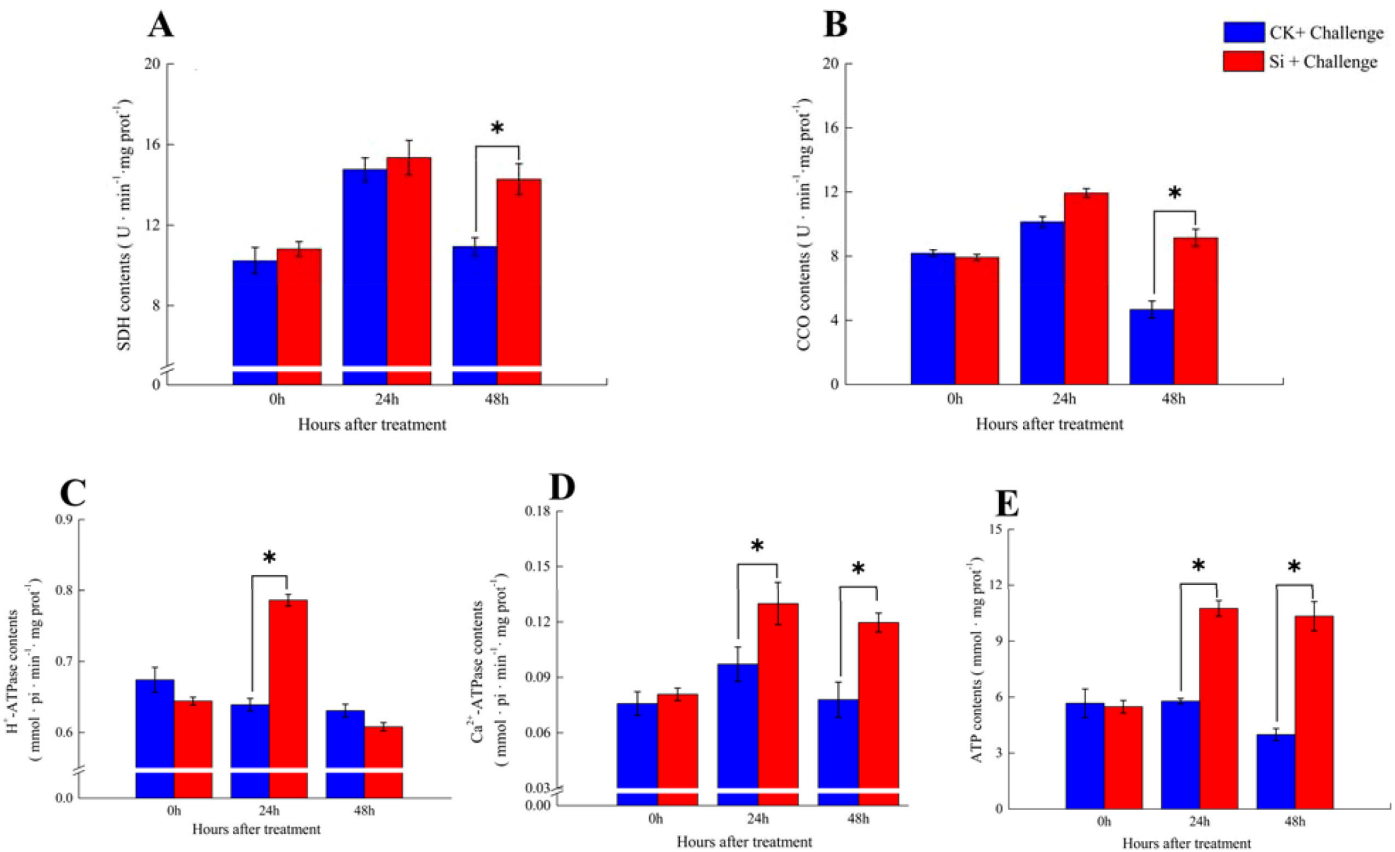
Effects of Si treatment followed by inoculation with *T. roseum* on the **(A)** SDH; **(B)** CCO; **(C)** H^+^-ATPase; **(D)** Ca^2+^-ATPase and **(E)** ATP contents; *, *p* < 0.05; CK + C, control check and pathogen challenge; Si + C, Si treatment and pathogen challenge. (Data represent mean ± SEM from n = 3 independent biological replicates).

The enzyme activities of SDH, CCO, H^+^-ATPase and Ca^2+^-ATPase displayed analogous dynamics across storage, with levels peaking prior to a gradual decrease. Regarding SDH, the Si treated group demonstrated a time-dependent enhancement compared to the infected control, showing a 3.99% increase at 24 hpi and a more pronounced 30.11% increase at 48 hpi of storage (**Figure 3A**, *p* < 0.05). Whereas regarding CCO, a prominent induction of CCO activity was observed in the Si-treated group compared to the infected control, showing a sustained elevation with a 16.75% increase at 24 hpi and a more pronounced 95.87% increase at 48 hpi of storage (**Figure 3B**, *p* < 0.05).

Si treatment induced a marked enhancement in H^+^-ATPase activity after 24 hpi, representing a 1.23-fold increase over the infected control (**Figure 3C**, *p* < 0.05). A marked elevation in muskmelon fruit Ca^2+^-ATPase activity was observed at 24 h, measuring 1.338 times the value of the infected group. The Ca^2+^-ATPase activity in Si-treated muskmelon fruit exhibited a marked induction over 24–48 hours, reaching levels 33.78% and 53.61% higher than the baseline at the respective time points (**Figure 3D**, *p* < 0.05). Moreover, Si treatment induced a prominent induction of ATP content, reaching levels 86.11% and 159.40% higher than the infected control at 24 h and 48 h, respectively (**Figure 3E**, *p* < 0.05).

### 3.4 DNA methylation profiling results

A comprehensive methylome analysis via WGBS was conducted at 24 hpi to investigate the alterations in DNA methylation patterns in response to Si treatment in *T. roseum*-inoculated muskmelon fruits. A total of 134, 283, 554 and 148, 499, 437 clean reads was generated. The average read depth for the Si-treated group and the infected-control ranged from 20.05 G to 19.47 G, with mapping rates of 85.70% to 87.53%.

**Figure 4:**
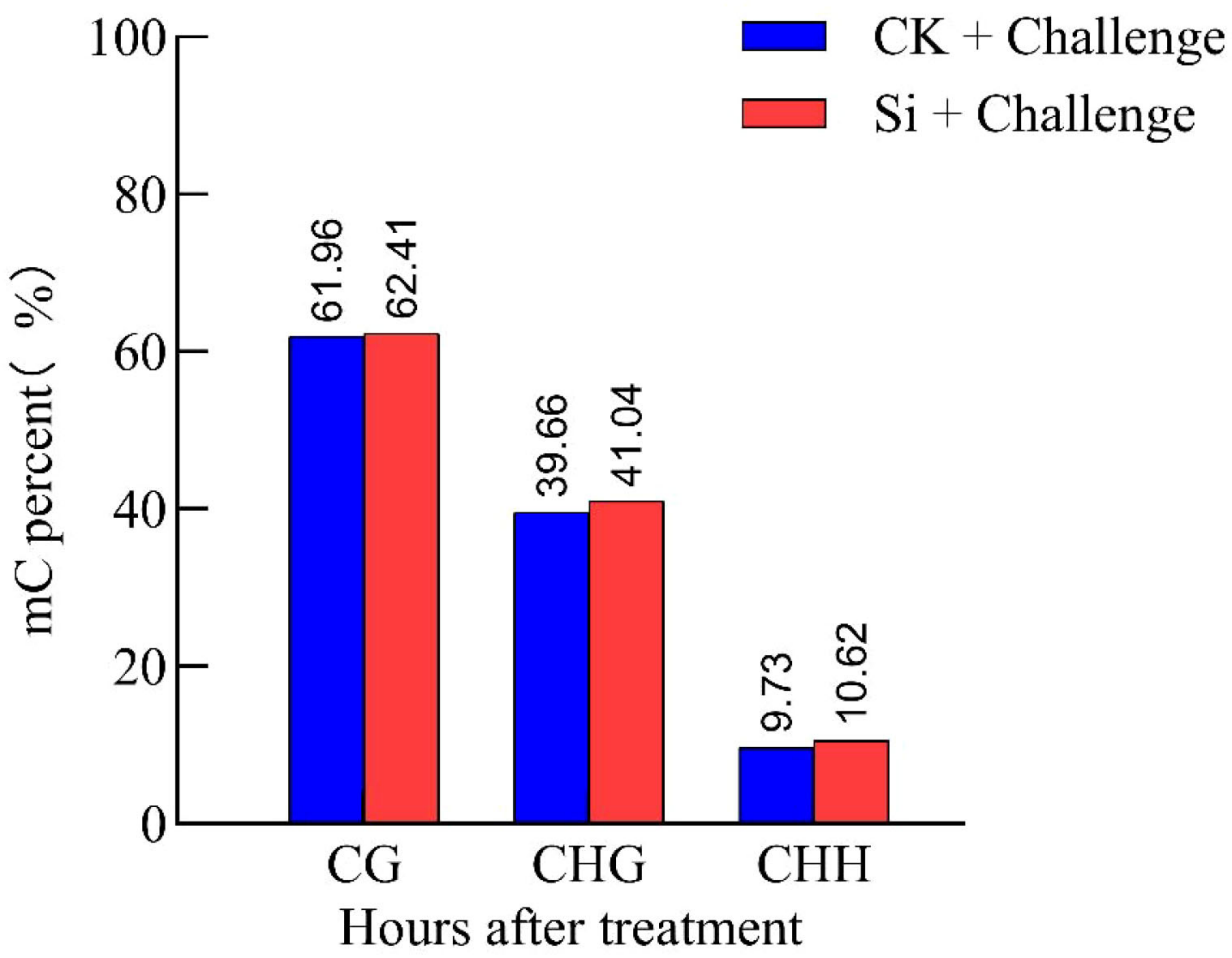
Global methylation pattern in the sequence contexts of CG, CHG, and CHH (H = A/T/C) in muskmelon fruits challenged with *T. roseum* after Si treatment by WGBS at 24 hpi; Each group consisted of 12 muskmelons, and the experiment was repeated three times.

Si application led to notably enhanced methylation ratios in all three sequence contexts—CG, CHG, and CHH. The methylation levels exhibited a distinct context-dependent distribution, with a decreasing gradient from CG (infected-control- 61.96%, Si-treated group- 62.41%) to CHG (infected-control- 39.66%, Si-treated group- 41.04%) to CHH (infected-control- 9.73%, Si-treated group- 10.62%) (**Figure 4**, H = A, T, or C).

**Figure 5:**
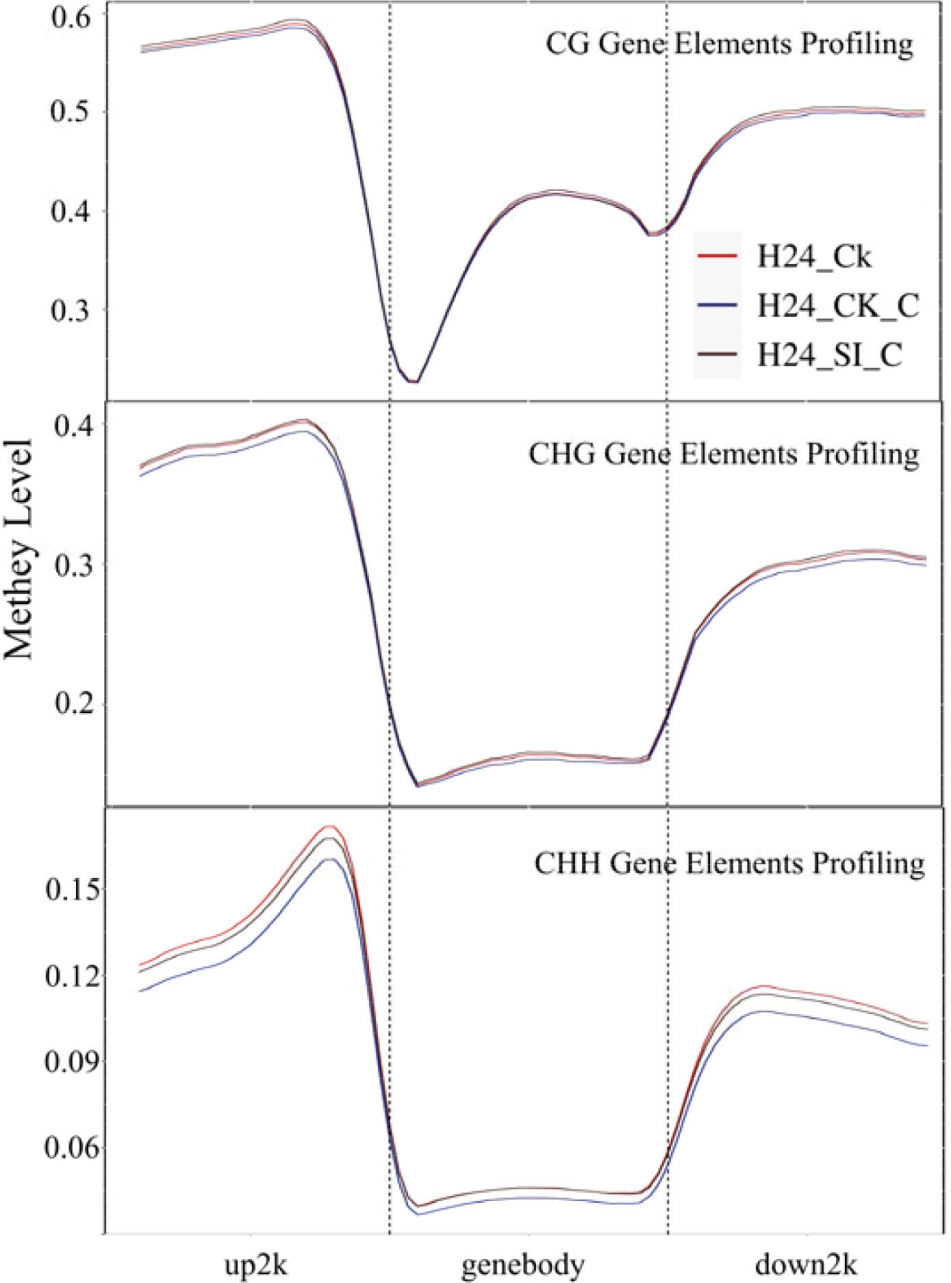
Changes in the levels of CG, CHG and CHH methylation in genebody, up2k and down2k regions; respectively in muskmelon fruit challenged with *T. roseum* after Si treatment by WGBS at 24 hpi.

A comparative analysis of methylation levels between the infected control and Si-treated groups revealed a general increase in methylated cytosines within gene bodies, up2k, and down2k regions. In addition, a distinct distribution pattern was observed, with the up2k regions displaying relative hypermethylation in contrast to the gene bodies (**Figure 5**, H = A, T, or C). A context-specific enhancement of DNA methylation was induced by Si treatment, showing a gradient of effect: CG context in the up2k regions were most affected, surpassing the response of CHG and CHH contexts (**Figure 5**, H = A, T, or C).

### 3.5 Identification of DMRs and DEGs

**Figure 6:**
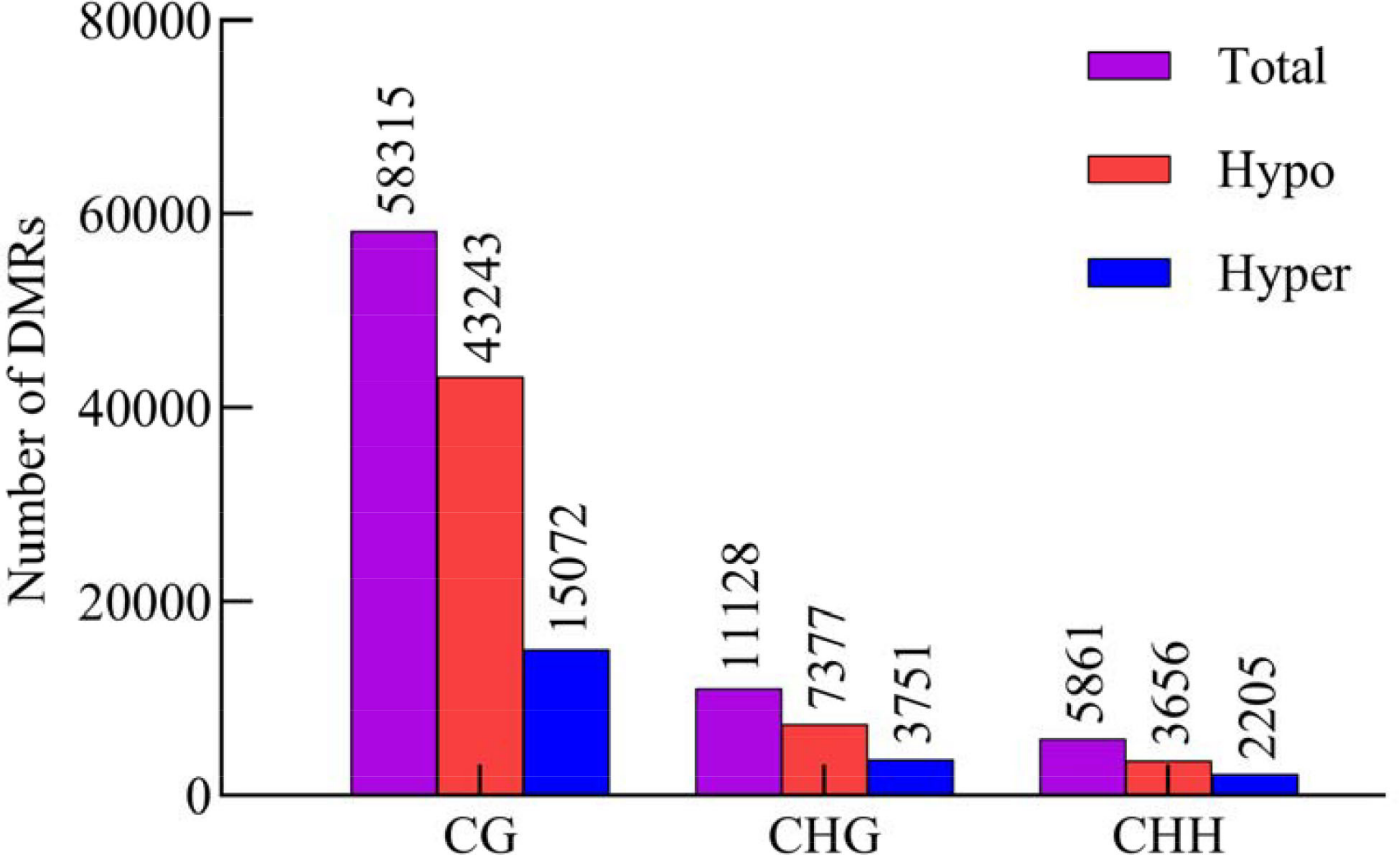
Total number of differentially methylated regions (DMRs); hypermethylated and hypomethylated regions identified between Si+C with CK+C at 24 hpi.

At 24 hpi, a comprehensive DMRs (DMRs, methylation differences % ≥ 10 or ≤ −10, *P* < 0.05) analysis showed that the CG context, which had the highest methylation density, also predominated in terms of DMR abundance, with 58, 315 regions, significantly more than those identified in CHG and CHH contexts (**Figure 6**). A total of 75, 304 DMRs were detected in Si treatment samples compared to the infected control, distributed as 54, 276 hypermethylated and 16, 528 hypomethylated regions (**Figure 6**). These findings imply that DNA methylation, particularly in the CG context, is preferentially involved in reprogramming the methylome during Si treatment. The CG context exhibited a significant skew towards hypermethylation, with hypermethylated DMRs being substantially over-represented compared to hypomethylated DMRs. A regional specificity was evident, with the majority of methylation variations predominantly localized to the up2k regions. A comparative analysis revealed that while CHG and CHH contained fewer DMRs than CG, the internal composition of DMRs in all contexts was skewed towards hypermethylation. The degree of skewness, quantified as the percentage excess of hyper- over hypomethylated DMRs, was 186.93% for CG, 96.67% for CHG, and 65.80% for CHH.

### 3.6 GO enrichment of genes associated with DMRs

**Figure 7:**
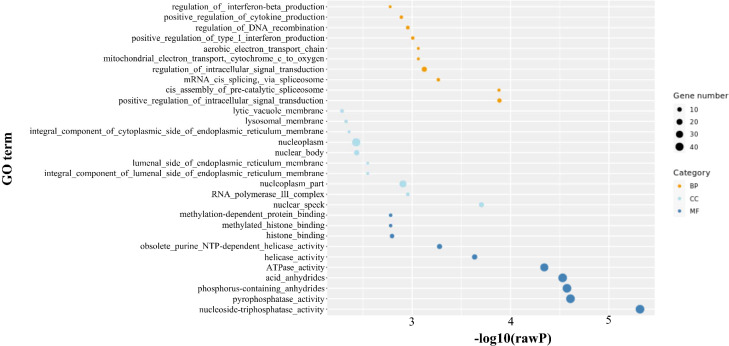
GO enrichment analysis of DMR-associated genes at 24 hpi; *p* values are corrected to –log10 (*p* values) ranging from 0 to infinity, and a lower *p* value (i.e., a greater –log10 (*p* value)) indicates a higher intensity; The top 10 GO enrichments of each categorizationis listed in descending order of the *p*-value.

Functional enrichment analysis indicated that DMGs were predominantly associated with regulation of interferon-beta production, positive regulation of cytokine production, and regulation of DNA recombination. Additionally, enrichment was observed in processes related to cellular growth and morphogenesis (unidimensional cell growth, cell morphogenesis, cell tip growth), photosynthesis (light reactions), developmental growth involved in morphogenesis, and interspecies interactions between organisms (**Figure 7**). Molecular function analysis indicated that hydrolase activity and cyclohydrolase activity were the most significantly over-represented terms among DMR-associated genes. Enrichment analysis of subcellular localization revealed that proteins linked to DMRs were significantly enriched in the nuclear speck, chloroplast stroma, plastid stroma, and cell tip (**Figure 7**).

### 3.7 Kyoto encyclopedia of genes and genomes pathway analysis

**Figure 8:**
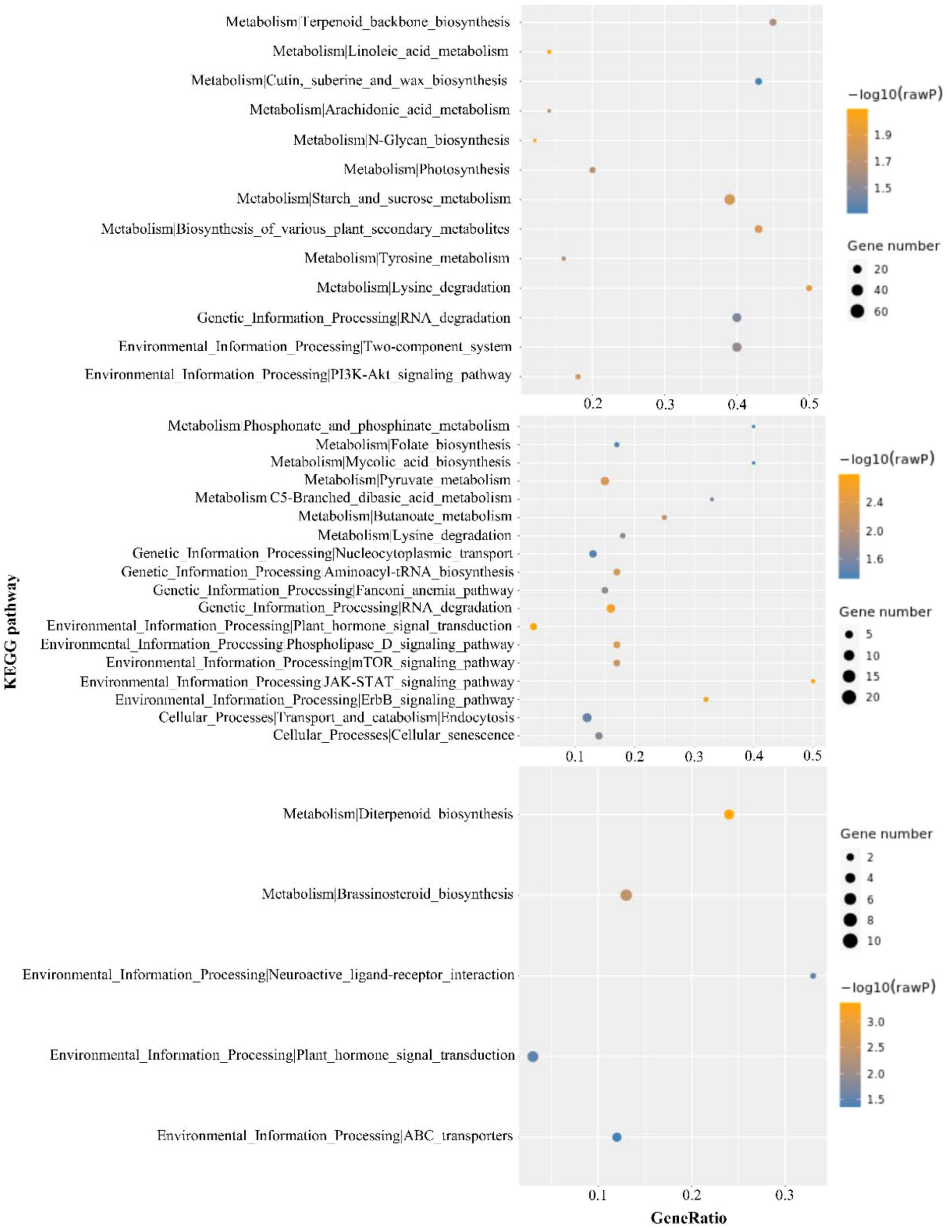
The top 20 KEGG pathways enriched for the common genes of DMGs and DEGs in CG, CHG and CHH context; respectively at 24 hpi; A pathway with *p* < 0.05 was considered significantly enriched.

Functional annotation and pathway enrichment analysis of the DEGs were carried out utilizing the KEGG database. Notably, in the context of methylated CG sites, the pathways showing the most pronounced enrichment signals included linoleic acid metabolism, N-Glycan biosynthesis, starch and sucrose metabolism, as well as metabolism and biosynthesis of various plant secondary metabolites. In addition, under CHG sites, pyruvate metabolism, RNA degradation and plant hormone signal transduction were enriched, while under CHH sites, diterpenoid biosynthesis and brassinosteroid biosynthesis exhibited significant enrichment (**Figure 8**).

### 3.8 Differentially expressed protein analysis

Tryptic digests were analyzed by LC-MS/MS, and the acquired data were queried against a specified protein sequence database. To generate a high-confidence protein list, the search results were filtered according to the following criteria: Sequest HT score > 0, the presence of at least one unique peptide, and the exclusion of all proteins identified in blank control runs. Following subcellular localization prediction, a subset of 267 proteins was assigned to the mitochondrial compartment.

Statistical significance was assessed for each mitochondrial protein across comparison groups using Student’s t-tests to derive fold change and p-values. The following dual criteria were applied to select differentially expressed proteins: |FC| > 1.2 (or FC < 5/6) accompanied by a *p*-value < 0.05. The results identified a total of 24 differentially expressed proteins, of which 5 were up-regulated and 19 were down-regulated (**Supplementary Table 1**). In addition, 71 DEGs were associated with the production of 24 DEPs in mitochondria. Of these, 14 were up-regulated and 57 were down-regulated (**Supplementary Table 2**).

### 3.9 GO and KEGG enrichment functional analysis of DEPs

**Figure 9:**
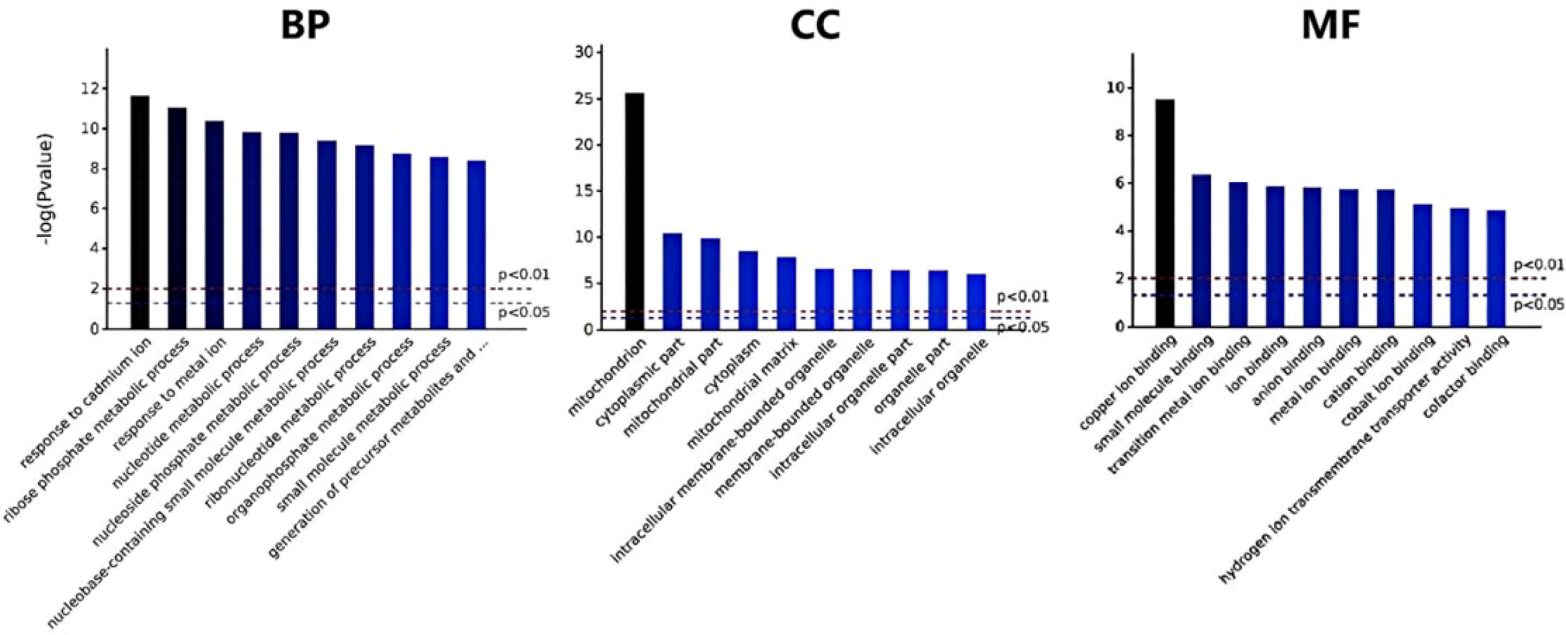
Differentially expressed proteins (DEPs) were subjected to GO functional annotation and enrichment analysis; *p* values are corrected to –log10 (*p* values) ranging from 0 to infinity, and a lower *p* value (i.e., a greater –log10 (*p* value)) indicates a higher intensity; A *p*-value < 0.05 was considered significant, and a *p*-value < 0.01 was considered highly significant; BP: Biological Process, CC: Cellular Component, MF: Molecular Function.

In-depth functional profiling of the DEPs was carried out through GO annotation and enrichment analysis, leveraging the OmicsBean integrated omics analysis cloud platform. The GO framework encompasses a tripartite classification: Biological Process, Cellular Component, and Molecular Function. Functional enrichment analysis demonstrated a significant over-representation of specific biological processes, notably response to cadmium ion, ribose phosphate metabolic process, and response to metal ion. A comprehensive visualization of the top 10 enriched processes is provided in **Figure 9**, highlighting their relative significance. Subcellular localization enrichment indicated that the differentially expressed proteins were predominantly enriched in mitochondria, cytoplasmic components, and the mitochondrial matrix. A rank-ordered list of the top 10 enriched cellular components is presented in **Figure 9**. Analysis of molecular functions demonstrated a significant enrichment for terms including copper ion binding, small molecule binding, and transition metal ion binding. A hierarchical list of the top 10 enriched molecular functions is provided in **Figure 9**.

**Figure 10:**
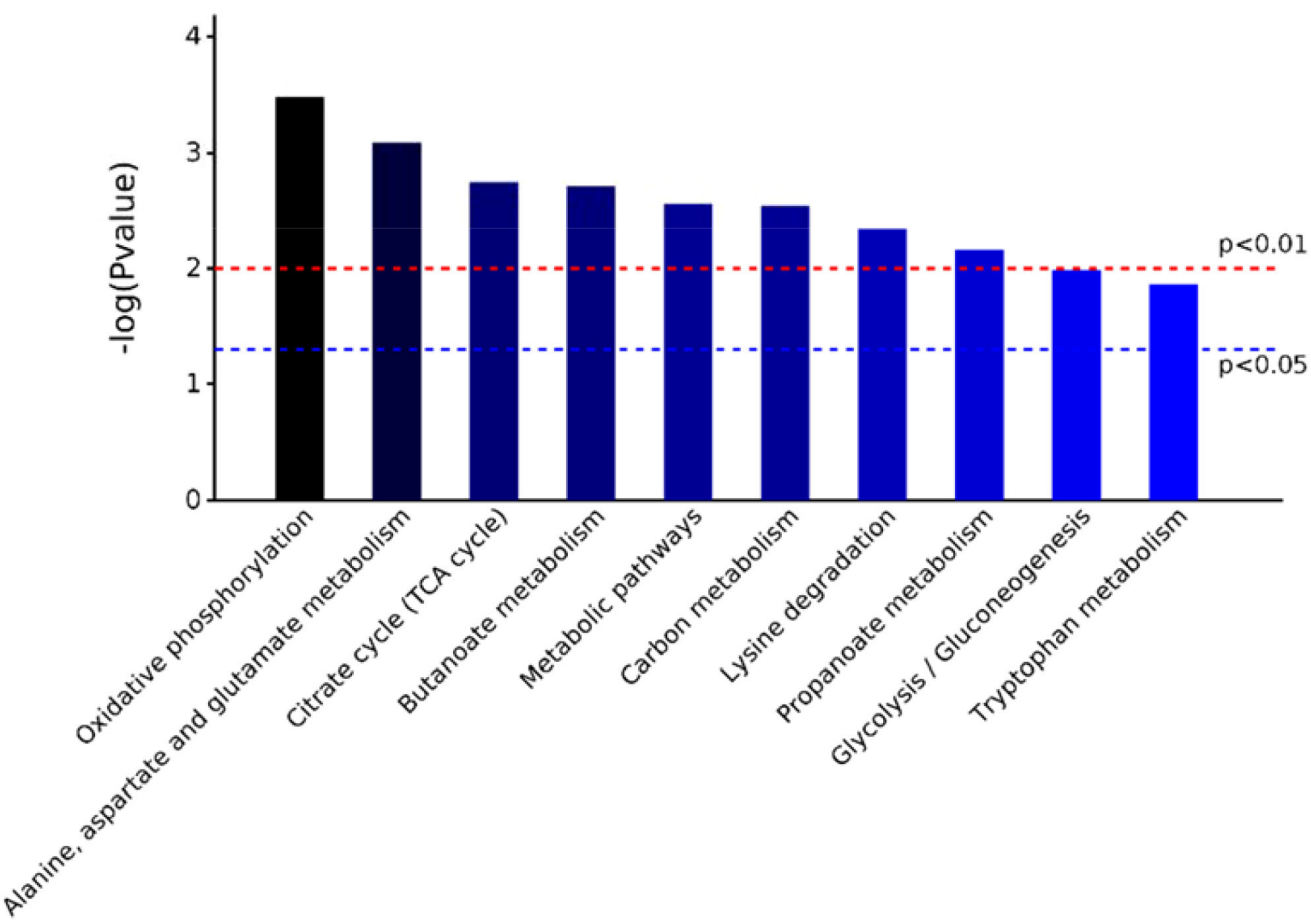
Top 10 KEGG pathways primarily involved by differentially expressed proteins; A *p*-value < 0.05 was considered significant, and a *p*-value < 0.01 was considered highly significant.

KEGG pathway enrichment analysis performed via the OmicsBean platform revealed Oxidative phosphorylation, Alanine, aspartate and glutamate metabolism, and the TCA cycle as top enriched pathways. Their ranking among the top 10 is detailed in **Figure 10**.

## 4 Discussion

Mitochondria are central to the regulation of cell death and immune responses (Gao et al., 2023). An adequate energy supply is a prerequisite for synthesizing defense-related compounds and maintaining membrane integrity. Physiological data revealed that *T. roseum* infection challenges the energy status of the fruit (Lyu et al., 2025a). However, Si treatment significantly alleviated this stress. We observed a marked elevation in ATP content in Si-treated fruits, reaching levels 86.11% and 159.40% higher than the infected control at 24 hpi and 48 hpi post-inoculation, respectively (**Figure 3E**).

ROS play a pivotal role in the processes by which plants respond to a diverse array of biotic and abiotic stresses (Torres, 2010). During the interaction between plants and pathogens, the generation of H_2_O_2_ and O_2_^•−^ can elicit the hypersensitive response, which constitutes a crucial disease resistance mechanism in plants. In the present study, Si treatment resulted in the accumulation of mitochondrial ROS during the early stages of the defense response in muskmelon fruits, indicating that Si treatment initiates the defense response of melon fruit against *T. roseum* infection (Ali et al., 2025). Si treatment enhances the defense response of muskmelon fruits through the promotion of ROS generation.

This energy burst was accompanied by upregulated enzymatic activities. Specifically, Ca^2+^-ATPase activity in Si-treated fruit was significantly induced, measuring 33.78% and 53.61% higher than the baseline at 24 hpi and 48 hpi (**Figure 3D**). The maintenance of high Ca^2+^-ATPase activity is crucial for regulating intracellular calcium signaling, which acts as a secondary messenger in plant immunity (Lu et al., 2021). These results align with previous findings that elicitors like acibenzolar-S-methyl and methyl jasmonate mitigate biotic and chilling stress by regulating energy metabolism (Ge et al., 2017; Jin et al., 2013). The ability of Si to sustain higher ATP levels and ATPase activity suggests that Si treatment prevents mitochondrial dysfunction, thereby ensuring sufficient energy currency for prolonged defense responses (Lyu et al., 2025b).

WGBS analysis yielded high-quality data (mapping rates > 85%), revealing that Si treatment induces widespread alterations in the epigenome. A key finding of this study is the context-specific enhancement of DNA methylation induced by Si. We observed that the CG context, particularly in the up2k (promoter) regions, was most responsive to the treatment, surpassing changes in CHG and CHH contexts (**Figure 5**). DNA methylation in promoter regions is traditionally associated with the repression of gene expression (Takatsuka and Umeda, 2015); However, recent evidence in plants suggests a more complex relationship where methylation can also stabilize transcriptional responses during stress (Yu et al., 2025). The hypermethylation observed in the promoter regions of specific genes in Si-treated melons may serve to fine-tune the expression of genes involved in energy metabolism and defense, preventing the energy drain often associated with hyper-immune responses or delaying senescence-related gene activation.

The correlation analysis between the methylome and transcriptome highlights the regulatory role of epigenetics in this system. The selection of the 24 h time point, guided by our methylation dynamics, proved critical in capturing the early transcriptional reprogramming events. The correspondence between DMRs and DEGs suggests that Si treatment recruits epigenetic machinery to modulate the transcription of nuclear-encoded mitochondrial genes (Lyu et al., 2025b). This likely results in the observed upregulation of proteins responsible for ATP synthesis and ion transport (Perveen et al., 2025).

The proteomic data strongly corroborated physiological and transcriptomic findings, providing a comprehensive view of the Si-induced resistance mechanism (Li Y. et al., 2023). We identified a subset of 267 proteins specifically assigned to the mitochondrial compartment, among which 80 differentially expressed proteins (DEPs) were consistently altered across treatment groups (defined by |FC| > 1.2 and *p* < 0.05). Functional profiling via GO and KEGG enrichment analyses revealed that Si treatment specifically targets metabolic machinery. The enrichment of biological processes such as ribose phosphate metabolic process and response to metal ion suggests that Si not only modulates fundamental energy metabolism but also triggers specific stress-responsive pathways akin to metal ion signaling (**Figure 8**). Crucially, KEGG pathway analysis highlighted Oxidative phosphorylation and the TCA cycle as the top enriched pathways among the DEPs. The upregulation of key enzymes within these pathways provides the molecular basis for the elevated ATP content and ATPase activities observed in Si-treated fruit (**Figure 8**).

Furthermore, the integration of multi-omics data revealed a functional convergence between the methylome and the proteome. Molecular function analysis indicated that hydrolase activity was significantly over-represented among both DMR-associated genes and DEPs. This overlap suggests that the Si-induced DNA methylation patterns in promoter regions effectively regulate the translation of specific hydrolytic enzymes. Consequently, the coordination between the epigenome and the proteome enables the melon fruit to mount a rapid and sustained defense response (Li et al., 2020; Vasantha-Srinivasan et al., 2025), characterized by reinforced mitochondrial function and enhanced metabolic flux.

In summary, this study demonstrates that Si treatment enhances resistance to *T. roseum* in harvested muskmelons by maintaining mitochondrial energy status, specifically via elevated ATP levels and Ca^2+^-ATPase activity (Li et al., 2020). Furthermore, we provide evidence that this physiological state is underpinned by dynamic epigenetic changes, specifically increased DNA methylation in the CG context of promoter regions (Talarico et al., 2025). These findings establish a link between inorganic nutrient application (Si), epigenetic regulation, and mitochondrial function, offering a theoretical basis for utilizing Si as a post-harvest treatment to extend the shelf life of melons.

## Data Availability

The original contributions presented in the study are publicly available. This data can be found here: CNCB, GRA043950.
